# Insights Following Implementation of an Exercise Intervention in Older Veterans with PTSD

**DOI:** 10.3390/ijerph16142630

**Published:** 2019-07-23

**Authors:** Michelle M. Pebole, Katherine S. Hall

**Affiliations:** 1 Geriatric Research, Education and Clinical Center, Durham VA Health Care System, Durham, NC 27705, USA; 2 Department of Medicine, Duke University, Durham, NC 27710, USA

**Keywords:** Post-traumatic stress disorder, physical activity, implementation, health promotion, aging, barriers, goal setting, motivation, exercise prescription

## Abstract

Individuals with post-traumatic stress disorder (PTSD) face numerous barriers to exercise. Little is known about behavioral strategies to promote participation in this population. This is a secondary analysis of individual barriers and goals, exercise prescription characteristics, and patient perceptions of a 12-week, community-based, randomized controlled exercise trial targeting older adults with PTSD, (*N* = 45; mean age = 68; male = 91%). The most common cited goals for participating included weight loss (65%) and increasing strength (65%). Exercise mode varied among those who completed the program (n = 37), with 14 (38%) using exclusively treadmill; eight (22%) using only bike, and 15 (41%) utilizing a combination. Patient-reported exercise duration and intensity progressively increased over the 12 weeks, and duration differed by mode of exercise. We observed high rates of attendance (84%) and completion (88%) to the program. Patient-reported barriers to attendance most often included health problems (62%) and medical appointments (55%). Participant responses to a program evaluation revealed high levels of satisfaction, preferences for group-based programs, and insights about the acceptability of the exercise environment (physical and social). This study is the first to report on goals, barriers, exercise prescription needs, and individual responses to supervised exercise training in a unique population, that is, older veterans with PTSD. Results of this study can inform future health promotion programs targeting older veterans with PTSD.

## 1. Introduction

The benefits of exercise on physical and psychological well-being are established [1,2,3]. However, research has just begun to examine exercise in individuals with mental health comorbidities. Post-traumatic stress disorder (PTSD) is prevalent among U.S. military service veterans and symptoms can persist over years, or even a lifetime [4]. The prevalence of PTSD is estimated to be upwards of 30% among Vietnam-era veterans [5]. High incidence of PTSD among aging veterans is troubling, considering the many negative long-term health consequences associated with chronic PTSD, including poor cardiometabolic health outcomes and psychological disorders [6,7,8,9]. PTSD is also negatively associated with physical activity; individuals diagnosed with PTSD are more physically inactive and spend more time in isolation, further increasing their risk for functional impairment and chronic health problems [1,2,3,10,11]. Older veterans engage in less physical activity than younger veterans, and thus are at greater risk for functional impairment and chronic disease [12]. 

Exercise among individuals with PTSD is a new research area. Preliminary evidence suggests regular exercise may improve PTSD symptoms [13,14,15,16] and may be attractive to older veterans who are less likely to initiate mental health treatment [17]. Recent work has also demonstrated beneficial effects of an acute exercise bout on mood and pain in individuals with PTSD [18,19]. Yet, veterans with PTSD often have lower participation and enrollment in health promoting programs targeting veterans [20]. Recent exercise intervention studies among individuals with PTSD provide some evidence of the acceptability and efficacy of these programs [14,21,22,23], but specific exercise motivations, barriers, and prescriptions often go unreported. Individuals with PTSD face additional barriers to engaging in exercise and health promoting behavior. These include pain, functional impairment, social isolation, and lack of motivation, among others [24,25,26]. Other implementation considerations in this population include exercise barriers and goals, as well as individualized exercise prescriptions and progression considerations. For example, among these medically complex patients with a high symptom burden, do we observe preferences for certain modes of activity, and do these characteristics impact individual exercise prescription and progression? This information is critical in considering efforts to tailor behavioral exercise interventions to increase adherence in this population and address functional impairments. Complete reporting of exercise interventions in this population is needed to translate and implement findings into clinical practice [27].

The Warrior Wellness program was a pilot randomized controlled trial of community-based exercise for older veterans with PTSD, and primary outcomes are reported elsewhere [28,29]. This study is an ancillary investigation of factors related to the exercise program, including programmatic factors related to exercise motivation, attendance barriers, exercise prescription and progression, and participant evaluations among those enrolled in the Warrior Wellness Program.

## 2. Materials and Methods 

### 2.1. Exercise Intervention

**Figure 1:**
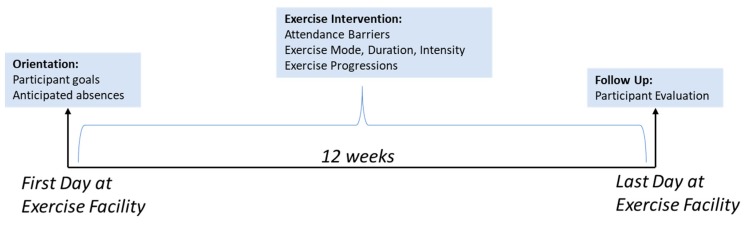
Study timeline and measures.

The Warrior Wellness program was a 12-week individualized, progressive, multi-component program developed according to guidelines for older adults [28,29,30]. Exercise prescription was developed by the exercise physiologist (EP) with input from each participant and guided by RPE ratings and participant feedback. Exercise sessions were offered three times a week. All exercises were modified as reported previously so they could be performed at several levels of difficulty, ranging from basic to advanced [28]. Each participant completed a daily exercise card (Appendix A) which recorded activities completed by the participant each day. Each week, the participant and EP reviewed everyone’s prescription and adjusted or progressed exercises based on physiologic responses and RPE ratings during exercise. Prior to the intervention, individuals went through an orientation visit, during which time they were familiarized with the gym layout and exercise environment, consulted with the EP, and identified individual goals. Figure 1 shows the study timeline and assessments that were completed.

The exercise sessions were held from 9:00–10:30 a.m. at a community-based fitness center located in close proximity to the Durham Veterans Affairs Medical Center (VAMC). Veterans exercised in a group, but each received their own prescription and completed it separately. Exercises utilized a mixture of free weights, cardiovascular machines, and weight machines. Each prescription was individualized by the EP and guided by RPE ratings and participant feedback. Review of exercise prescriptions occurred weekly. Individualized prescriptions usually consisted of a brief warm up, 8–10 different strengthening exercises, followed by aerobic exercise. During each exercise session, the participant filled out their individual exercise card to track their progress. Program attendance was recorded daily by the EP and reasons for missed exercise sessions were recorded by the EP on an attendance log. After the 12-week intervention was completed, veterans completed a post-program evaluation. 

A detailed description of study methods is published elsewhere [28]. All study procedures were approved by the Durham VAMC Institutional Review Board. Written informed consent was obtained for all procedures. This pilot randomized controlled exercise trial for older veterans with PTSD was registered in a public registry (ClinicalTrials.gov identifier NCT02295995).

### 2.2. Setting and Participants

The parent trial examined the feasibility and efficacy of supervised exercise versus a wait-list control. Clinically significant improvements in PTSD and related conditions (sleep, depressive symptoms, and mental health-related quality of life) were reported following 12 weeks of supervised exercise, compared to wait-list usual care [29]. The study reported here is an ancillary investigation of the prescription and implementation of the exercise program in this clinical trial. As such, only those veterans who enrolled and completed the exercise program were included in these analyses, and no between-arm comparisons were required. Fifty-four participants were randomized: 36 to exercise (PA), 18 to wait-list usual care (WL). Of the 18 randomized to WL, 9 transitioned into the supervised exercise program after the initial 12-week period; bringing the total number of participants who engaged in exercise to 45. 

Inclusion and exclusion criteria are detailed elsewhere [28]. In short, study participants included veterans age ≥60 years who met Diagnostic and Statistical Manual of Mental Disorders (DSM-V) [31] criteria for PTSD. The Clinician-Administered PTSD Scale for DSM-V (CAPS-5) diagnostic interview [32] was used by the researchers to determine final eligibility. Exclusion criteria included lifetime history of any psychiatric disorder with psychotic features and active alcohol or substance abuse, among others. 

### 2.3. Measures

All measures were completed by either the participant or the EP. Data were collected at several timepoints including an orientation session before the exercise began, daily on exercise logs during the exercise session, or after intervention completion (Figure 1). Measures are detailed below: 

Participant Goals: 

Exercise Goals: Exercise participants were asked to complete a goal-setting worksheet with the EP at the orientation session prior to initiating exercise. The worksheet instructed the participant to identify two or three self-selected goals using S.M.A.R.T. (Specific, Measurable, Agreed-upon, Realistic, Time-based) principles [28]. Veterans discussed goals with the EP, adjusted goals as necessary to fit the S.M.A.R.T. principles, and recorded them as part of a behavioral contract.

Exercise Prescription: 

Exercise intensity, mode of exercise, and exercise duration were recorded on the participant’s exercise card.

Exercise Intensity: Exercise intensity was measured using rating of perceived exertion (RPE; 0–10 scale). RPE for the aerobic exercise session was recorded on the exercise card following completion of the entire aerobic exercise bout [29]. RPE is a valuable and valid measure of exercise intensity in populations such as older adults in which heart rate measures of exercise intensity are inaccurate due to heart rate limiting medications. Because the exercise group began as sedentary, intensity targets were an RPE of 2 or 3 (“light” and “moderate”) at the beginning of the intervention. As the intervention went on, level of intensity targets increased to an RPE of 3 to 7 (“moderate,” somewhat hard,” and “hard”). 

Mode of Aerobic Exercise: To accommodate functional limitations, participants were offered 4 aerobic exercise modalities: Recumbent stepper (SCIFIT), stationary bike, treadmill, and elliptical. The EP aimed to get each participant to utilize the treadmill by the end of 12 weeks, as it offers both aerobic and balance training, and higher intensities. For those participants with mobility and/or pain limitations, stationary bike and recumbent stepper were offered as a lower intensity option. The EP suggested aerobic exercise modes to the participants, but ultimately they self-selected their machine. Participants could also split their aerobic exercise between two machines. The exercise participant recorded their mode or modes of exercise daily on their exercise card. 

Aerobic Exercise Duration: Exercise duration was recorded each day by the participant on his/her exercise card. The progressive exercise program aimed to have participants up to 30 min of continuous aerobic exercise by week 6. During the first 6 weeks, the EP set suggested duration goals for each participant based on the recorded RPE and duration from the previous week.

Attendance Related Measures: 

Attendance: The EP tracked daily attendance in an attendance log.

Anticipated barriers: During the orientation session, the EP asked participants, “what are some barriers to attendance and completion of exercise sessions that you foresee?”. The EP recorded participant responses on the orientation sheet.

Reasons for Absence: Recorded in conjunction with attendance. Following an absence (expected or unexpected), the EP inquired about the reason for the absence when the patient returned to exercise. 

Acceptability:

Participant Perceptions: Upon completion of the exercise program, participants were asked to complete a formal program evaluation. This form included targeted and open-ended questions about their experience with the program, perceptions of the exercise environment (physical and social), and preferences for the content and delivery of similar, future exercise programs. Example items include, “I felt comfortable in the exercise setting (1 (strongly disagree)–4 (strongly agree)).” 

### 2.4. Statistical Analyses

A detailed analysis of pre-program goals and barriers to exercise, exercise prescription elements, participation rates, and participant feedback of the program was conducted. Participants who enrolled and/or completed the exercise program (*n* = 45) were the focus of these analyses. 

Goals and Barriers: Goals and barriers were qualitatively examined for major themes and reported as the percentage of participants who identified goals/barriers in specific domains.

Exercise mode: Three types of exercise modes were identified for analysis: Exclusively treadmill, exclusively stationary bicycle, and a combination of machines. The combination group consisted of veterans who utilized a mix of machines including the elliptical, bike, treadmill, or recumbent stepper. The percentage of participants in each group was calculated. 

Exercise Intensity: Average RPE was calculated for all participants at week 1, week 6 (mid-way through the program), and week 12 (end of the exercise program). 

Duration: Average duration of aerobic activity was calculated at weeks 1, 6, and 12. 

Participant Evaluations: Results were evaluated using descriptive statistics. 

Retention and Adherence: Retention rate was characterized by the number of participants who completed the 12-week assessments. Number of sessions attended divided by number sessions offered was used to calculate attendance percentage.

## 3. Results

### 3.1. Participant Characteristics 

Participant characteristics at baseline are shown in Table 1.

The average age of participants was 67.5 years (range 60–76 years). These veterans were primarily Vietnam-era (Nov 1955–1975; 88.9%). The average duration of symptoms was 45 years. The sample was mostly male (91.1%) and African American (88.9%), with significant health burden (average 3.9 comorbid health conditions). The mean baseline BMI was 30 kg/m^2^, and 82.2% of the sample met criteria for overweight or obese. PTSD symptom severity in this sample was high, and average scores of the PHQ-9 revealed moderate levels of depressive symptoms. Participant characteristics of the entire sample, by arm, have been reported previously [29].

### 3.2. Participant Goals

Goals were collected from every veteran who began the exercise intervention (*n* = 42); however, one veteran did not identify any goals, and one veteran had data missing, leaving *n* = 40. The most common types of goals for participating in the exercise program included increased strength (65%), weight loss (65%), improving overall health (33%), improving balance (30%), and improving cardiovascular health (23%). 

### 3.3. Exercise Prescription 

Our analysis of the mode of aerobic exercise among participants who completed the exercise program (*n* = 37) showed that 8 (22%) exclusively used the bicycle, 14 (38%) exclusively used the treadmill, and 15 (41%) used a combination of machines. Of the 15 veterans in the combination group, 7 utilized the bike and treadmill, 6 used the treadmill and elliptical, 1 used the recumbent stepper and bike, and 1 used the arm ergometer and bike. 

Participant RPE ratings reflected the progressive intensity of the program, beginning at ‘moderate’ (avg. RPE @ week 1 = 3), increasing to ‘somewhat hard’ by mid-way (avg. RPE @ weeks 2 to 6 = 4), and remaining somewhat hard/heavy (avg. RPE = 4–5) for weeks 7 to 12. 

The program aimed to work participants up to 30 continuous min of aerobic exercise by week 6. Our analysis of the duration of aerobic exercise among those who completed the exercise program (n = 37) showed that average aerobic exercise duration at week 1 = 10 min, at week 6 = 25 min, at week 12 = 28 min. Exercise duration at 12 weeks differed by mode of exercise: the bike-only group averaged shorter duration (20 min) than the treadmill-only group (27 min) and the mixed-method group (33 min). An exploratory analysis comparing the bike-only to the treadmill-only and mixed mode groups showed participants who opted for bike-only had significantly higher (*p* < 0.05) BMI and poorer physical function. No differences were shown between these groups for age, number of chronic conditions, or pain.

### 3.4. Adherance and Attendance Barriers 

The completion rate among those who initiated exercise was high (88%). Forty-five initiated exercise and 3 withdrew from participation prior to the first visit. Out of the 42 participants that started the exercise intervention, 5 (12%) dropped out before completing the entire 12-week protocol. Of the 37 veterans who completed the exercise intervention, the average attendance rate was 84% (range: 51% to 100%). 

Prior to beginning the exercise program, participants were asked to report any anticipated barriers to regularly participating in the 12-week exercise program. Over half (55%) of veterans reported that they did not anticipate any barriers. Medical appointments (20%), mood disturbances (7%), and pain (7%) were the most commonly cited anticipated barriers.

Patient-reported reasons for a missed exercise session largely fell into three domains: Health problems including acute illness, chronic illness flare-up, mental health, and pain (62%); medical appointments that conflicted with exercise session days/times (55%); and travel conflicts (31%). Six participants had missing data for this variable and were not included in the analysis. 

### 3.5. Program Evaluations 

Post-program assessments revealed high levels of satisfaction, with all respondents indicating they would recommend this program to other veterans with PTSD. Over 90% of participants reported improvements in physical and mental health at the end of the program. Respondents also indicated that the exercise prescription (progression and intensity) was appropriate for their physical condition; all participants reported that they enjoyed the exercises. Relative to the physical and social environments, participants reported being comfortable in the exercise setting (97%), confidence in the ability of the EP (100%), and social connection with both the EP and other participants. Over 80% of participants indicated interest in combining exercise with dietary counselling in future interventions, as well as opening program enrollment to significant others. The majority (60%) of respondents reported satisfaction with the program duration of 12 weeks, though 40% indicated a program of longer duration would be ideal. When asked about time of day, participants indicated morning exercise times were convenient. Regarding program delivery, respondents indicated slight preference for programs that offered a mix of home- and facility-based sessions (54%) over exclusively facility-based programs (46%). None of the respondents indicated interest in exclusively home-based programs.

Detailed responses are shown in Table 2. 

## 4. Discussion

Although the benefits of exercise for mental health are well-known, individuals with PTSD are rarely targeted for health promotion interventions. As a result, little is known about exercise promotion strategies in this clinical population. This is the first study to report findings on program design, psychosocial factors, and patient perceptions of a supervised exercise program for older adults with PTSD. Results across goal setting, exercise barriers, and program design indicate individual tailoring is essential to developing a safe, effective, and engaging exercise program in this population.

### 4.1. Participant Goals 

The pre-exercise orientation session was used to explore participant goals and provided valuable insights into an individual’s motives for participating in the program. This information was used by the study staff to counsel participants about realistic outcome expectations and to review elements of the exercise program that corresponded to their individual goals. Consistent with previous work in exercise and mental illness [35], many participants cited wanting to improve health as a goal for the intervention. Although Warrior Wellness did not personalize exercises based on specific goals, the progressive nature of the exercise intervention naturally addressed improving strength and function in health. Several participants also endorsed goals related to weight loss. However, physical activity in the absence of dietary intervention, plays only a small role in weight loss [36], and thus, Warrior Wellness did not aptly address weight loss goals identified by participants. This is significant when considering the impact of outcome expectations on exercise adherence and maintenance, with unrealistic expectations associated with higher rates of attrition [37,38]. The orientation session was an opportunity for the EP to provide patient education about realistic expectations (e.g., weight loss) and physiologic changes (strength, health) with 12 weeks of exercise training. Information gathered on participant goals showed participants were highly motivated to improve strength and overall health, revealing opportunities for delivering future patient-centered exercise programs. Participant feedback also suggests that supervised exercise plus supplemental dietary education would be widely embraced by this population. The formal assessment of patient goals and related behavioral counselling were critical elements of this program. People with mental illness are generally receptive to becoming more physically active, but often lack motivation [39,40]. We recommend including a pre-exercise assessment of participant goals in future exercise interventions targeting this population. 

### 4.2. Participation Barriers 

Data collected on exercise barriers revealed new information as well as opportunities to increase adherence in future interventions. Previous work has reported lethargy, lack of motivation, and weight gain as frequently cited barriers to exercise in individuals with mental illness [35,41,42]. However, we are unaware of any studies reporting on PTSD-specific barriers to supervised exercise. Reported anticipated barriers included conflicting medical appointments, mood disturbances, and pain. Of note, we observed discrepancy in the patient-reported anticipated barriers to participating versus actual recorded absences during the program. Over half of the veterans reported that they anticipated no barriers to regularly participating in the program for 12 weeks, once again reflecting unrealistic expectations. Most absences were due to health problems and/or medical appointments, which is consistent with the clinical profile of individuals with PTSD that includes high rates of health care utilization and medically complex comorbid conditions (e.g., depression, chronic pain). High program attendance revealed facility-based exercise programs are feasible in this population. However, given reported barriers did include high frequencies of scheduling conflicts, it is worthwhile to consider addressing these barriers in future programming. 

Participant responses on the program evaluation revealed opportunities that may address the complex needs of this population while also promoting physical activity. For example, interest in future programs that included a mixture of home- and facility-based sessions was high. Options for home-based sessions would provide veterans increased flexibility to attend needed medical appointments without sacrificing exercise involvement. These findings are consistent with previous research indicating programmatic flexibility is important in promoting adherence to exercise interventions in mental health populations [43,44,45]. However, previous studies in older adults with and without mental health conditions demonstrate that supervised exercise programs experience better adherence and greater improvements in fitness and health [46,47]. High program attendance in this trial further emphasizes that supervised programs are feasible and impactful in this population. Our program evaluation data reveal participants received social support from exercise staff and other participants. Previous work in mental health has demonstrated the importance of social environment in exercise settings, emphasizing accountability, trust, and encouragement from the environment and staff [40,41,48,49,50]. It is therefore important for future programs to include skilled exercise supervision as well as behavioral expertise in the form of individualized social support. 

### 4.3. Tailoring 

Medical complexities in this population, particularly musculoskeletal injuries and chronic pain, necessitated individualized functional assessments and tailoring throughout the intervention. These older adults with PTSD had high levels of comorbid conditions and functional impairment. Thus, exercise prescriptions often varied according to individual capabilities. For instance, study staff often offered alternative options and/or adaptations for veterans who experienced high levels of knee or hip pain. Patient-centered exercise prescription allowed for full engagement among veterans with varying levels of functionality. Consistent with previous findings [43,44,45], our data indicate that rigid prescription is not feasible in this population. Physiologists need to be able to tailor exercise modes, duration and intensities to ensure engagement throughout interventions. Despite diversity in exercise prescriptions, evaluations revealed the programming was challenging and well-liked; our exercise intervention data also indicate that it is possible to write diverse, patient-centered prescriptions while still uniformly progressing in intensity. These individualized exercise prescription efforts promoted adherence by (1) allowing participants to self-select exercises they enjoyed, and (2) allowing veterans to select exercises that adapted to daily fluctuations in pain, discomfort, or mobility. Our results underscore the importance of highly skilled and experienced exercise physiologists who can aptly address diverse physical functional needs and prescribe appropriate exercise progressions and routines in this population [27,39,48]. 

### 4.4. Additional Future Considerations

This intervention reports on aspects of individualization from the Warrior Wellness program, a supervised exercise intervention for older veterans with PTSD. Exclusion criteria in this trial included comorbid psychiatric conditions and current substance abuse. These conditions are common in individuals with PTSD. Therefore, the generalizability of this study is limited. Researchers recruited a predominately minority population that accurately reflects the demographics of older veterans with PTSD [51,52]. However, researchers recognize these findings could differ among other groups of military veterans with PTSD (Caucasians, women), [53]. Expansion of the sample may reveal more detailed and nuanced reports of barriers, goals, and prescriptions in this population. Additionally, there are several significant aspects of behavior change theory that were not assessed in this study, and that may have impacted exercise participation, including but not limited to social support, enjoyment, self-efficacy, and exercise environment [54]. Previous work has demonstrated the importance of the social and physical environments to exercise [40,41,48,49,50], which are significant when considering future implementation efforts. Additionally, population-specific aspects of intervention delivery such as trauma-informed exercise settings represent an understudied area of implementation efforts in PTSD populations. 

## 5. Conclusions

Individuals with PTSD are not traditionally targeted for health promotion interventions; this report investigates exercise promotion strategies for this clinical population. Warrior Wellness was a successful intervention, with high rates of attendance, adherence, and satisfaction. Individualization efforts by the study staff contributed to the success of the intervention. Findings suggest motivations and barriers among aging veterans with PTSD do have similarities to the general population and other mental health conditions [35,40,42]. All participants reported high satisfaction with the program, and over 90% reported improvements in physical and mental health upon completion of the program. This demonstrates that exercise is an important and beneficial health promotion strategy in this population. Our study findings are expected to inform future tailoring efforts in individuals with PTSD. 

## Figures and Tables

**Table 1:** Participant Characteristics (*N* = 45).

Variable	* M(SD)
Age (yrs)	67.5 (3.3) Range: 60–76
Sex (male), n (%)	41 (91.1%)
Race (AA), n (%)	40 (88.9%)
Comorbidities	3.9 (1.7)
Overweight / Obese, n (%)	37 (82.2%)
BMI (kg/m^2^)	30.0 (5.6) Range: 21–47
Education, ≥some college, n (%)	28 (62.2%)
Depression (PHQ9; 0–24) [33]	10.2 (5.2) 49% ≥ 10 score
PTSD symptoms (PCL-5; 0–80) [34]	41.3 (14.5) Range: 13–70

* Mean (Standard Deviation).

**Table 2:** Responses to Program Evaluation.

Item	Mean (SD)	Median (Min–Max)	*N* (%) *
**Overall Review**			
** Please circle your overall reaction to Warrior Wellness (1 = excellent; 4 = poor)	1.1 (0.4)	1 (1–2)	37 (100%)
** Please circle your overall reaction to the instructors (1 = excellent; 4 = poor)	1 (0.2)	1 (1–2)	37 (100%)
** Overall, how do you feel physically after completing the Warrior Wellness exercise program (1 = excellent; 4 = poor)	1.6 (0.6)	2 (1–3)	35 (95%)
** Overall, how do you feel mentally after completing the Warrior Wellness program (1 = excellent; 4 = poor)	1.6 (0.6)	2 (1–3)	34 (92%)
**Additional Comments**			
I plan to continue doing the exercises on my own (1 = strongly disagree; 4 = strongly agree)	3.4 (0.7)	4 (2–4)	32 (86%)
I would recommend Warrior Wellness to other Veterans with PTSD (1 = strongly disagree; 4 = strongly agree)	3.8 (0.4)	4 (3–4)	37 (100%)
**Exercise Program**			
The exercises were too hard given my physical condition (1 = strongly disagree; 4 = strongly agree)	1.6 (0.9)	1 (1–4)	3 (8%)
The exercise intensity progressed at an appropriate pace for me (1 = strongly disagree; 4 = strongly agree)	3.6 (0.6)	4 (2–4)	36 (97%)
I enjoyed the selected exercises (1 = strongly disagree; 4 = strongly agree)	3.6 (0.5)	4 (3–4)	37 (100%)
**Exercise Environment**			
The time Warrior Wellness was held was convenient (1 = strongly disagree; 4 = strongly agree)	3.7 (0.5)	4 (2–4)	36 (97%)
I felt comfortable in the exercise setting (1 = strongly disagree; 4 = strongly agree)	3.7 (0.5)	4 (2–4)	36 (97%)
I feel emotionally close to others in the exercise group (1 = strongly disagree; 4 = strongly agree)	3.2 (0.7)	3 (1–4)	26 (87%)
**Instructors**			
The instructors helped me adapt the exercises to fit my level of ability (1 = strongly disagree; 4 = strongly agree)	3.9 (0.3)	4 (3–4)	37 (100%)
I feel emotionally close to the instructors (1 = strongly disagree; 4 = strongly agree)	3.5 (0.8)	4 (1–4)	28 (93%)
**Future Study Considerations**			
I wish the program would have addressed diet/eating habits (1 = strongly disagree; 4 = strongly agree)	3.2 (0.7)	3 (2–4)	30 (81%)
I wish my spouse or significant other could have participated in the program (1 = strongly disagree; 4 = strongly agree)	3.4 (0.7)	3 (2–4)	30 (81%)
What type of exercise setting do you prefer	(54%) Mix of home- and facility-based (46%) Facility-based (0%) Home-based		
How do you like to exercise?	(46%) In a group, doing my own routine (22%) Alone (16%) In a group, instructor-led class		
Is the Warrior Wellness program length adequate? (12 weeks)	(40%) Not long enough (60%) Yes, 12 wks was adequate		

Response items ranged from (1 (strongly disagree)–4 (strongly agree)). * *N* (%) = those who rated ≥3 OR ≤2 for reverse coded items, indicating strong endorsement of the item. ** Item responses are reverse coded.

## Appendix A

**Daily Exercise Card.**
